# Sex Differences in Self-Rated Health and Cardiovascular Disease Events

**DOI:** 10.1001/jamanetworkopen.2026.4129

**Published:** 2026-04-01

**Authors:** Maneesh Sud, Feng Qiu, Olivia Haldenby, Sunjidatul Islam, Peter C. Austin, Douglas Manuel, Dean Eurich, Padma Kaul, Michelle M. Graham, Mina Madan, Thao Huynh, Erica S. Spatz, Harindra C. Wijeysundera, Dennis T. Ko

**Affiliations:** 1 Schulich Heart Program, Sunnybrook Health Sciences Centre, University of Toronto, Toronto, Ontario, Canada; 2 Temerty Faculty of Medicine, University of Toronto, Toronto, Ontario, Canada; 3 ICES, Toronto, Ontario, Canada; 4 Institute of Health Policy, Management and Evaluation, University of Toronto, Toronto, Ontario, Canada; 5 Ottawa Hospital Research Institute, Ottawa, Ontario, Canada; 6 Department of Family Medicine, University of Ottawa, Ottawa, Ontario, Canada; 7 School of Public Health, University of Alberta, Edmonton, Canada; 8 Department of Medicine, University of Alberta, Edmonton, Canada; 9 Department of Medicine, Faculty of Medicine and Health Sciences, McGill University, Montreal, Quebec, Canada; 10 Department of Medicine, Division of Cardiology, McGill University Health Centre, Montreal, Quebec, Canada; 11 Section of Cardiovascular Medicine, Yale School of Medicine, Yale University, New Haven, Connecticut

## Abstract

**Importance:**

Clinical risk factors, lifestyle factors, and social determinants of health have established sex-specific associations with new-onset cardiovascular disease (CVD). However, little is known about the role of patient-reported health measures in individuals without CVD.

**Objective:**

To determine whether self-rated health is independently associated with the development of CVD and whether this association differs by sex.

**Design, Setting, and Participants:**

This retrospective cohort study included community-dwelling adults enrolled in the Ontario Health Study from March 1, 2009, to December 31, 2017, with no prior CVD or active cancer. Outcome ascertainment was via linkage to administrative databases for follow-up outcomes to March 31, 2024, and data were analyzed from January 5, 2025, to January 16, 2026.

**Exposure:**

Excellent, very good to good, and fair to poor self-rated health.

**Main Outcomes and Measures:**

The primary outcome was CVD events (hospitalization for myocardial infarction, stroke, heart failure, and cardiovascular death). Cause-specific hazard models with an interaction between self-rated health and sex were adjusted for age, traditional risk factors, lifestyle factors, social determinants of health, and family history of CVD.

**Results:**

The cohort consisted of 170 197 participants (104 789 [61.6%] women; median age, 48 [IQR, 36-58] years) followed up for a median of 12.1 (IQR, 12.0-12.3) years. Fair to poor health was reported in 11 661 women (11.1%) and 6381 (9.8%) men; very good to good health, in 75 819 (72.4%) women and 47 865 (73.2%) men; and excellent health, in 17 309 (16.5%) women and 11 162 (17.1%) men. After adjustment, poorer self-rated health was associated with a higher rate of CVD in both sexes. Compared with excellent self-rated health, the fully adjusted hazard ratios (HRs) for fair to poor self-rated health were 2.08 (95% CI, 1.80-2.40) in women and 1.45 (95% CI, 1.29-1.64) in men; the HRs for very good to good self-rated health compared with excellent health were 1.26 (95% CI, 1.11-1.43) in women and 1.04 (95% CI, 0.94-1.14) in men (*P* < .001 for interaction).

**Conclusions and Relevance:**

In this cohort study of individuals without CVD, as many as 1 in 10 rated their health as fair to poor. Fair to poor self-rated health was an independent risk factor associated with new CVD, and its relative hazard was greater in women than in men. These findings support the use of a simple self-assessment of health to aid in risk stratification in the primary prevention of CVD.

## Key Points

**Question:**

Is self-rated health associated with the development of cardiovascular disease (CVD), and does this association differ by sex?

**Findings:**

In this cohort study of 170 197 participants, poorer self-rated health was associated with higher rates of new CVD, with a greater relative hazard in women compared with men.

**Meaning:**

These findings support the use of a simple self-assessment of health to aid in risk stratification in the primary prevention of CVD.

## Teaser

This cohort study assesses whether self-rated health is associated with development of cardiovascular disease among adults in Ontario, Canada, and whether the association differs by sex.

## Introduction

An individual’s global rating of their health status is a measure of how physical, social, and psychological health impact their well-being.^1,2^ This broadly encompasses symptom burden, functional status, health-related quality of life, interpersonal relationships, social cohesion, optimism, and a sense of purpose. Guidelines encourage the use of patient-reported measures of health to facilitate shared decision-making to improve care and outcomes.^1^ While self-rated health status has been incorporated into multiple health-related quality of life instruments that are used to quantify disease severity and treatment effects,^3,4^ its role in cardiovascular disease (CVD) risk assessments remains undefined. Indeed, preventative guidelines emphasize the need for patient-centric decision-making; however, they provide little guidance on whether a patient’s rating of their own health should be considered.^5,6,7,8^

Sex-specific factors that enhance CVD risk are increasingly used in clinical practice to identify individuals most likely to benefit from preventive treatment.^9^ While epidemiologic studies suggest self-rated health status is associated with the development of CVD,^10,11,12^ how the association differs between women and men is unknown. An extensive body of literature suggests that many physical, social, lifestyle, and psychological determinants of health confer a higher risk of CVD in women relative to men.^13,14,15,16,17,18,19^ Thus, we hypothesize that self-rated health may also be associated witha higher risk of new CVD in women when compared with men independently of known CVD determinants. Hence, we used a large, contemporary primary prevention cohort of women and men in Ontario, Canada, to determine whether the association between self-rated health and outcomes differed between sexes and whether this was independent of traditional cardiovascular risk factors, behavioral and lifestyle factors, social determinants of health, and family history of CVD.

## Methods

### Data Sources

The Ontario Health Study (OHS) is the largest prospective, contemporary longitudinal study in Canada in which more than 200 000 community-dwelling Ontario residents were voluntarily enrolled to study the epidemiology of and risk factors contributing to chronic diseases.^20^ Recruitment was primarily through invitation e-mails and letters, advertising, primary care clinicians, other stakeholders, incentive programs, and friends and family referrals. Baseline health-related questionnaires were completed at enrollment, while a subset of participants attended community-based study centers to undergo blood, urine, and physical measurements. Using unique encoded identifiers, anonymized linkages were performed to provincial administrative and laboratory databases such as (1) the Canadian Institute for Health Information Discharge Abstract Database to determine prior as well as longitudinal outcome hospitalization events,^21^ (2) the Ontario Registrar General Database for cause of death, (3) the Ontario Laboratories Information System for laboratory data,^21,22^ (4) the Ontario Drug Benefit Database to ascertain residence in a long-term care facility, and (5) the Immigration, Refugees and Citizenship Canada Permanent Residents Database for recent immigration status. All data analyses were completed at ICES (formerly known as the Institute for Clinical Evaluative Sciences), and Research Ethics Board approval was obtained through the Sunnybrook Health Sciences Centre. All participants provided informed consent. The study followed the Reporting of Observational Studies in Epidemiology (STROBE) reporting guideline for cohort studies.

### Study Cohort

Ontario residents with valid health insurance numbers who completed the baseline questionnaire between March 1, 2009, and December 31, 2017, were included. The index date was set as the date of questionnaire completion. Individuals younger than 18 years or older than 105 years were excluded. Individuals who had been hospitalized for myocardial infarction, stroke, heart failure, peripheral arterial disease, or atrial fibrillation as well those undergoing any inpatient or outpatient coronary revascularization procedure in the 20 years prior to the index date were excluded to create a representative cohort of individuals free of CVD and eligible for primary prevention assessments.^21^ Additionally, individuals receiving chemotherapy for cancer in the prior year, those with any history of metastatic cancer, moderate to severe liver disease, or dementia, those receiving chronic dialysis, or residents of long-term care facilities in the past 5 years were excluded since these chronic illnesses may impact self-rated health, limit life expectancy, or limit the potential long-term benefit of CVD preventative therapies.^23,24^ Finally, individuals with missing data for self-rated health were excluded.

### Self-Rated Health

Self-rated health was obtained from the baseline questionnaire. Individuals were asked “How would you rate your general health?” and provided the options: excellent, very good, good, fair, poor, or prefer not to answer. Individuals who did not select a response or indicated prefer not to answer were considered missing. For regression analysis, responses were simplified to 3 categories: excellent, very good to good, and fair to poor.

### Baseline Cardiovascular Risk Factor Domains

We defined several domains of CVD risk determinants that were subsequently used for imputation and risk adjustment. These included (1) age; (2) cardiovascular risk factors and enhancers, such as traditional factors (high blood cholesterol level, diabetes, hypertension, obstructive sleep apnea, body mass index, waist-to-hip ratio 0.90 or greater in men and 0.85 or greater in women, levels of serum total cholesterol, high-density lipoprotein cholesterol, triglycerides, hemoglobin A_1c_, fasting glucose, estimated glomerular filtration rate, and systolic blood pressure), inflammatory diseases that are known CVD risk enhancers included in prevention guidelines (psoriasis, rheumatoid arthritis, and lupus), and mental health disorders (depression and anxiety); (3) lifestyle and behavioral factors (smoking, fruit and vegetable consumption, sleep duration, physical activity level as measured by the international physical activity questionnaire); (4) social determinants of health (self-reported race and ethnicity, level of education attained, annual household income, active employment, immigration to Canada within the 20 years prior to the index date, and marital status); (5) geographic residence (residence within 1 of 3 previously defined regions in Ontario that are associated with ambulatory care service utilization and CVD rates^25^ or rural residency); and (6) genetic factors (a family history of CVD). Self-reported race and ethnicity (categorized as Black, East Asian, Latin, Middle Eastern, South Asian, Southeast Asian, White, multiracial, or other race or ethnicity) were included to provide a description of the demographic profile of the cohort while recognizing their role as a social construct. Furthermore, they were included in adjusted models given their association with incident CVD.^26^ Administrative coding definitions used to define baseline variables are provided in eTable 1 in Supplement 1.^21,27,28,29,30,31,32,33,34^

### Outcomes

The primary outcome was a CVD event defined by cardiovascular death or hospitalization for myocardial infarction, stroke, or heart failure, which aligns with outcomes used for risk stratification in contemporary primary prevention.^35,36^ Secondary outcomes included components of the primary outcome and death from any cause. Hospitalization outcomes were ascertained using validated algorithms.^21,27,28,29^ Cause-specific mortality outcomes were ascertained from the Ontario Registrar General Database and validated administrative algorithms.^30^ Patients were followed up until the outcome of interest, death, or March 31, 2024.

### Statistical Analysis

The rate of CVD events was reported per 1000 person-years of follow-up in the entire cohort and stratified by sex and baseline self-rated health status. Cumulative incidence curves were generated using the Aalen-Johansen estimator, which accounts for the competing event of noncardiovascular death.^37^ Differences in cumulative incidence curves across self-rated health categories were assessed in each sex using the Gray test.^38^ For regression analysis, multiple imputation with 30 complete datasets was used to account for missing data (eFigure 1 in Supplement 1).^39,40^ The imputation model included sex, self-rated health, the aforementioned domains of CVD risk, outcome indicators, and the cumulative hazards for CVD and death.^41^ In each complete dataset, the association between sex and self-rated health was assessed using a logistic regression model for excellent vs nonexcellent health. Models were adjusted for the domains described above. Measures of association were reported as odds ratios (ORs) along with 95% CIs. The association between self-rated health and CVD events was estimated using cause-specific hazards models in the setting of competing risks of non-CVD death (and a Cox proportional hazards regression model for all-cause mortality).^42^ Unadjusted models included an interaction term between self-rated health and sex. Models were then sequentially adjusted for each of the aforementioned CVD risk domains. Measures of association were reported as hazard ratios (HRs) with 95% CIs. For secondary outcomes, time to-event analysis was repeated after multiple imputation was performed separately using outcome indicators and cumulative hazards for each component of the primary outcome and death. We performed additional sensitivity analyses. Time-to-event analysis was repeated after multiple imputation was performed using 5 rather than 3 categories of self-rated health and excluding individuals with anxiety or depression. We used Rubin rules to pool measures of association and their SEs across the 30 complete datasets.

Data were analyzed from January 5, 2025, to January 16, 2026. All analyses were conducted using SAS, version 9.4 (SAS Institute Inc). Two-sided *P* < .05 indicated statistical significance.

## Results

### Study Cohort

Among 183 545 individuals who completed the baseline questionnaire, 7146 (3.9%) with prior CVD, 3579 (1.9%) with severe comorbidities or residents of long-term care facilities, and 2623 (1.4%) with missing data for self-rated health were excluded (eFigure 2 in Supplement 1). The final study cohort consisted of 170 197 individuals. The median age was 48 (IQR, 36-58) years with 104 789 (61.6%) women and 65 408 (38.4%) men. Among those who reported their race and ethnicity, 1.3% (95% CI, 1.3%-1.4%) were Black, 4.1% (95% CI, 4.0%-4.2%) were East Asian, 0.8% (95% CI, 0.8%-0.9%) were Middle Eastern, 3.4% (95% CI, 3.3%-3.5%) were South Asian, 1.1% (95% CI, 1.0%-1.1%) were Southeast Asian, 80.9% (95% CI, 80.7%-80.9%) were White, 4.5% (95% CI, 4.4%-4.6%) were multiracial or multiethnic, and 3.0% (95% CI, 2.9%-3.1%) reported other race or ethnicity. Baseline characteristics stratified by sex are reported in the Table. The median age in women was 47 (IQR, 34-56) years while the median age in men was 50 (IQR, 38-61) years. Women were less likely to have high cholesterol levels, diabetes, and hypertension, yet more likely to have depression and anxiety. More women than men consumed greater than 5 servings of fruits and vegetables daily, had low levels of physical activity, and had an annual household income less than $50 000, while fewer had a bachelor’s or graduate level education and were married or living with a partner.

**Table.** Baseline Characteristics^a^

Characteristic	Proportion, % (95% CI)			*P* value
	Overall (N = 170 197)	Women (n = 104 789)	Men (n = 65 408)	
Demographic				
Age, median (IQR), y	48 (36-58)	47 (34-56)	50 (38-61)	<.001
Social determinants of health				
Race and ethnicity				
Black	1.3 (1.3-1.4)	1.4 (1.3-1.4)	1.3 (1.2-1.4)	<.001
East Asian	4.1 (4.0-4.2)	3.3 (3.2-3.4)	5.4 (5.2-5.6)	
Latin	0.8 (0.7-0.8)	0.7 (0.7-0.8)	0.9 (0.8-0.9)	
Middle Eastern	0.9 (0.8-0.9)	0.7 (0.6-0.7)	1.2 (1.1-1.3)	
South Asian	3.4 (3.3-3.5)	2.3 (2.2-2.4)	5.2 (5.1-5.4)	
Southeast Asian	1.1 (1.0-1.1)	1.0 (1.0-1.1)	1.1 (1.1-1.2)	
White	80.9 (80.7-81.1)	82.3 (82.0-82.5)	78.7 (78.3-79.0)	
Multiracial	4.5 (4.4-4.6)	5.0 (4.9-5.2)	3.7 (3.6-3.9)	
Other^b^	3.0 (2.9-3.1)	3.4 (3.2-3.5)	2.5 (2.4-2.6)	
Recent immigrant	8.2 (8.1-8.3)	6.7 (6.5-6.8)	10.7 (10.4-10.9)	<.001
Annual household income <50 000 CAD$/y	27.7 (27.4-27.9)	30.3 (30.0-30.6)	23.5 (23.1-23.8)	<.001
University bachelor’s or graduate education level	44.5 (44.3-44.7)	42.6 (42.3-42.9)	47.6 (47.2-48.0)	<.001
Actively employed	67.6 (67.3-67.8)	67.1 (66.9-67.4)	68.2 (67.8-68.6)	<.001
Traditional risk factors				
High blood cholesterol level	19.0 (18.8-19.2)	14.4 (14.2-14.6)	26.4 (26.0-26.7)	<.001
Diabetes	7.6 (7.5-7.7)	6.0 (5.9-6.2)	10.1 (9.9-10.3)	<.001
Hypertension	21.9 (21.7-22.1)	18.8 (18.6-19.1)	26.8 (26.4-27.1)	<.001
Obstructive sleep apnea	6.6 (6.4-6.7)	4.3 (4.1-4.5)	10.2 (9.9-10.4)	<.001
Elevated waist to hip ratio	61.8 (61.5-62.1)	57.5 (57.1-58.0)	68.6 (68.1-69.1)	<.001
Depression	11.4 (11.3-11.6)	14.2 (14.0-14.4)	7.0 (6.8-7.2)	<.001
Anxiety	10.7 (10.3-11.1)	13.0 (12.5-13.5)	6.9 (6.5-7.3)	<.001
Rheumatoid arthritis	3.3 (3.2-3.4)	3.4 (3.3-3.5)	3.1 (3.0-3.2)	.003
Lupus	0.4 (0.3-0.4)	0.5 (0.5-0.6)	0.1 (0.1-0.1)	<.001
Psoriasis	4.8 (4.7-4.9)	4.7 (4.6-4.8)	4.9 (4.7-5.1)	.04
Total cholesterol level, median (IQR), mg/dL	190 (164-216)	194 (168-220)	183 (156-209)	<.001
High-density lipoprotein cholesterol level, median (IQR), mg/dL	56 (46-67)	61 (51-71)	49 (40-58)	<.001
Triglyceride level, median (IQR), mg/dL	110 (68-162)	103 (63-154)	122 (78-175)	<.001
HbA_1c_ level, median (IQR), %	5.6 (5.2-6.1)	5.6 (5.1-6.1)	5.7 (5.2-6.2)	<.001
Fasting glucose level, median (IQR), mg/dL	91.9 (81.1-106.3)	90.1 (79.3-102.7)	95.5 (84.7-109.9)	<.001
Estimated glomerular filtration rate, median (IQR), mL/min	97 (85-109)	98 (86-110)	95 (83-107)	<.001
Systolic blood pressure, median (IQR), mm Hg	117 (106-128)	114 (103-125)	122 (112-133)	<.001
BMI ≥30	23.5 (23.3-23.7)	23.8 (23.5-24.1)	23.0 (22.6-23.3)	
Lifestyle and behavioral factors				
Active daily smoker	10.6 (10.4-10.7)	11.0 (10.8-11.2)	9.9 (9.6-10.1)	<.001
≥5/d Servings of fruits and vegetables	46.3 (46.1-46.6)	53.6 (53.3-53.9)	34.8 (34.4-35.2)	<.001
7 to <9 h/d Sleep	64.4 (64.2-64.6)	64.8 (64.5-65.1)	63.8 (63.4-64.2)	<.001
Low physical activity level	29.0 (28.8-29.3)	30.0 (29.7-30.3)	27.5 (27.1-27.8)	<.001
Marital status				
Divorced	7.6 (7.5-7.7)	9.4 (9.2-9.6)	4.7 (4.6-4.9)	<.001
Married and or living with partner	67.4 (67.2-67.6)	63.4 (63.1-63.7)	73.7 (73.4-74.1)	
Separated	3.9 (3.8-4.0)	4.4 (4.2-4.5)	3.1 (3.0-3.3)	
Single or never married	18.3 (18.1-18.5)	19.2 (18.9-19.4)	16.9 (16.7-17.2)	
Widowed	2.8 (2.7-2.9)	3.6 (3.5-3.7)	1.5 (1.4-1.6)	
Residence				
Region in a high–CVD risk region	14.0 (13.8-14.1)	14.8 (14.6-15.0)	12.5 (12.3-12.8)	<.001
Rural residence	9.6 (9.4-9.7)	10.4 (10.2-10.5)	8.3 (8.0-8.5)	<.001
Genetic factors (family history of CVD)	39.9 (39.7-40.1)	40.1 (39.8-40.4)	39.6 (39.2-39.9)	.03

Abbreviations: BMI, body mass index (calculated as weight in kilograms divided by height in meters squared); CAD$, Canadian dollars; CVD, cardiovascular disease; HbA_1c_, hemoglobin A_1c_.

SI conversion factors: To convert cholesterol to mmol/L, multiply by 0.0259; glucose to mmol/L, multiply by 0.0555; HbA_1c_ to a proportion of total hemoglobin, multiply by 0.01; triglycerides to mmol/L, multiply by 0.0113.

^a^ Results are pooled across multiply imputed 30 datasets using Rubin rule.

### Self-Rated Health Status

Baseline characteristics across self-rated health categories are presented in eTable 2 in Supplement 1. Most cardiovascular risk factors and enhancers, lifestyle and behavioral factors, and social determinants of health were associated with self-rated health. For instance, the proportion with high cholesterol levels, diabetes, hypertension, obesity, low levels of physical activity, low fruit and vegetable consumption, active smoking history, low income, unemployment, and lower education level increased as self-rated health status worsened.

Despite having fewer cardiovascular risk factors and enhancers, more women indicated they had fair to poor health compared with men (11 611 [11.1%] vs 6381 [9.8%]), while fewer women rated their health as very good to good (75 819 [72.4%] vs 47 865 [73.2%]) or excellent (17 309 [16.5%] vs 11 162 [17.1%]) (*P* < .001). After adjusting for differences in age, cardiovascular risk factors and enhancers, and behavioral and lifestyle factors between sexes, women were more likely to not rate their health as excellent (OR, 1.18; 95% CI, 1.16-1.21). This persisted when additionally accounting for social determinants of health, residence, and family history (OR, 1.16; 95% CI, 1.14-1.19).

### Self-Rated Health Status and CVD Outcomes

**Figure 1.**
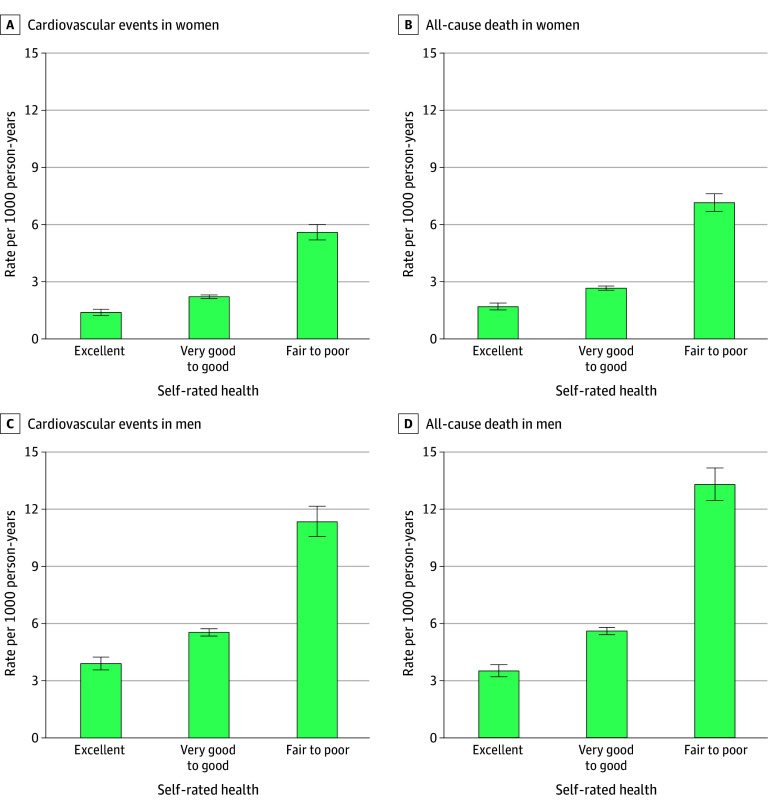
Bar Graphs of Rates of Cardiovascular Events and Death by Sex The unadjusted rate per 1000 person-years and corresponding 95% CIs (error bars) of cardiovascular events and death is depicted by self-rated health status in women and men.

**Figure 2.**
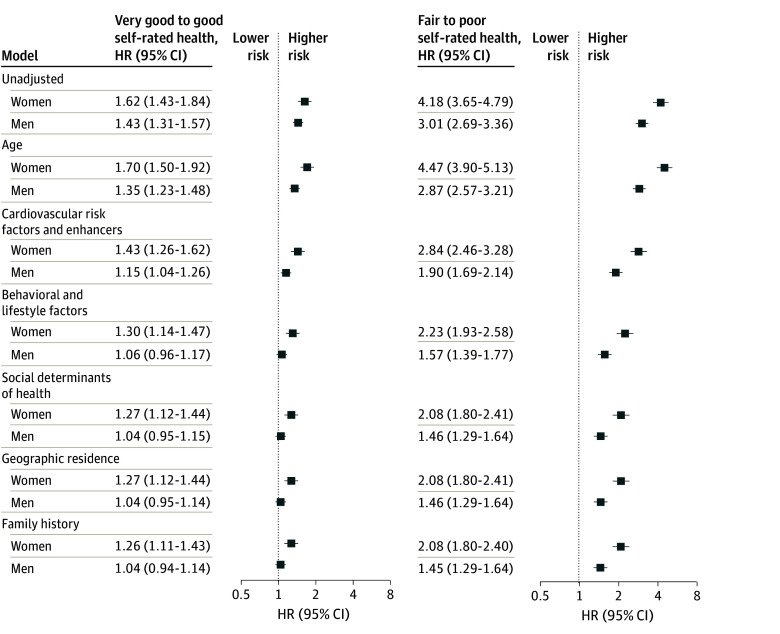
Forest Plot of the Association Between Self-Rated Health and Cardiovascular Outcomes by Sex Models were sequentially adjusted for age, clinical factors, lifestyle and behavioral factors, social determinants of health, geographic residence, and family history of cardiovascular disease. *P* < .001 for the interaction between sex and self-rated health status in all models. The reference category is excellent self-rated health. HR indicates hazard ratio.

After a median of 12.1 (IQR, 12.0-12.3) years of follow-up, the rate of CVD in women was 2.4 (IQR, 2.3-2.5) per 1000 person-years with 3018 CVD events and 2124 women dying from noncardiovascular causes. The rate of CVD in women for excellent self-rated health was 1.4 (95% CI, 1.2-1.5) per 1000 person-years; very good to good, 2.2 (95% CI, 2.1-2.3) per 1000 person-years; and fair to poor, 5.6 (95% CI, 5.2-6.0) per 1000 person-years (Figure 1). The cumulative incidence curves for CVD events in women differed across categories of self-rated health (*P* < .001), were nonoverlapping with early separation, and demonstrated a gradual rise in events (eFigure 3 in Supplement 1). In women, relative to excellent self-rated health, the unadjusted HR for very good to good self-rated health was 1.62 (95% CI, 1.43-1.84) and for fair to poor self-rated health was 4.18 (95% CI, 3.65-4.79). In men, the rate of CVD was 5.8 (95% CI, 5.6-5.9) per 1000 person-years with 4395 CVD events and 2239 men dying from noncardiovascular causes. The rates of CVD events increased from 3.9 (95% CI, 3.6-4.2) per 1000 person-years for excellent to 5.5 (95% CI, 5.3-5.7) per 1000 person-years for very good to good and 11.3 (95% CI, 10.5-12.1) per 1000 person-years for fair to poor self-rated health. The cumulative incidence curves in men also differed across categories of self-rated health (*P* < .001) (eFigure 3 in Supplement 1). After adjusting for sex, the HRs were greater for women compared with men for very good to good self-rated health (1.62 [95% CI, 1.43-1.84] vs 1.43 [95% CI, 1.31-1.57]) and fair to poor self-rated health (4.18 [95% CI, 3.65-4.79] vs 3.01 [95% CI, 2.69-3.36]) (*P* < .001) (Figure 2).

**Figure 3.**
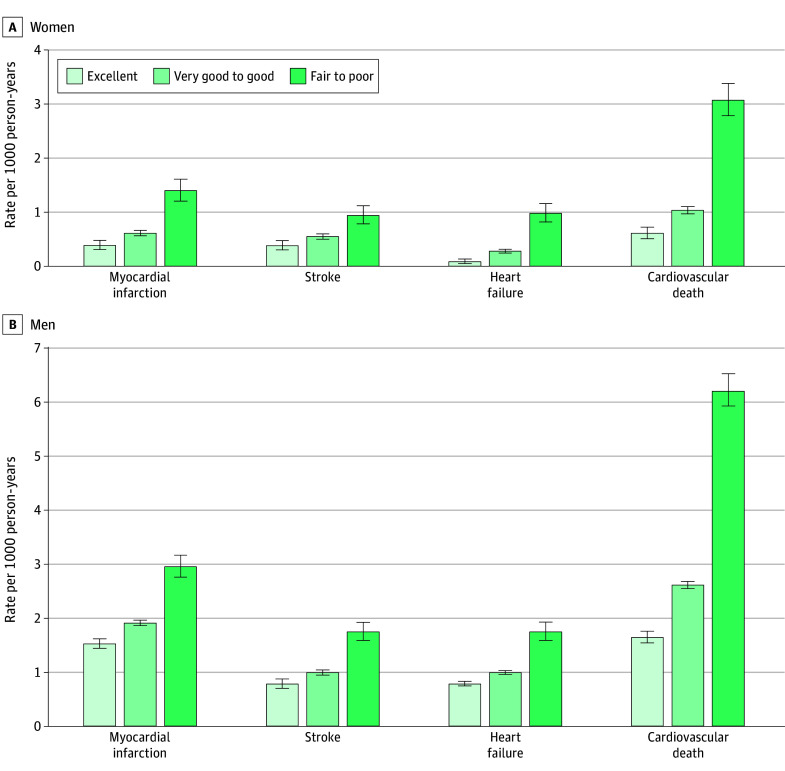
Bar Graphs of Rates of Secondary Cardiovascular Outcomes by Sex The unadjusted rate per 1000 person-years and corresponding 95% CIs (error bars) of myocardial infarction, stroke, heart failure, and cardiovascular death is depicted by self-rated health status in women and men.

Sequential adjustment for age, cardiovascular risk factors and enhancers, and behavioral and lifestyle factors partly attenuated but did not eliminate the sex-specific association between self-rated health and CVD. After additional adjustment for behavioral and lifestyle factors, the HRs remained greater for women compared with men for very good to good (1.30 [95% CI, 1.14-1.47] vs 1.06 [95% CI, 0.96-1.17]) and fair to poor self-rated health (2.23 [95% CI, 1.93-2.58] vs 1.57 [95% CI, 1.39-1.77]) (*P* < .001) (Figure 2 and Figure 3). Additional adjustment for social determinants of health and geographic residence resulted in a smaller attenuation of the association. After additional adjustment for family history of CVD, the fully adjusted HRs were once again greater for women compared with men for very good to good self-rated health (1.26 [95% CI, 1.11-1.43] vs 1.04 (95% CI, [0.94-1.14]) and fair to poor self-rated health (2.08 [95% CI, 1.80-2.40] vs 1.45 [95% CI, 1.29-1.64]) (*P* < .001).

### Secondary Outcomes and Sensitivity Analyses

**Figure 4.**
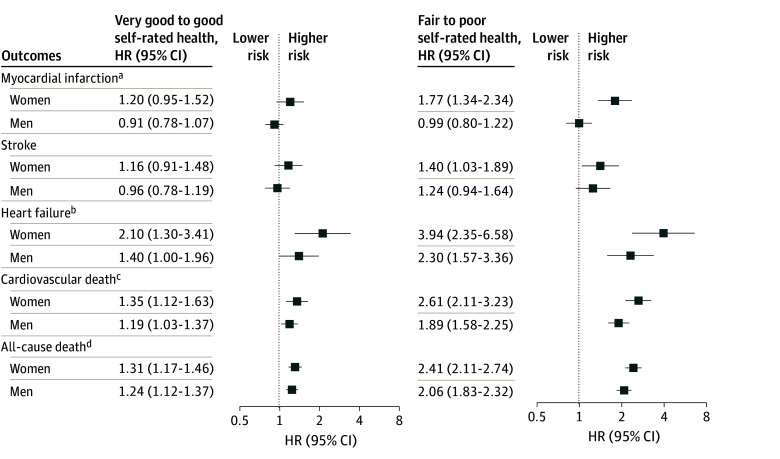
Forest Plot of Association Between Self-Rated Health and Secondary Outcomes by Sex Models were adjusted for age, clinical factors, lifestyle and behavioral factors, social determinants of health, geographic residence, and a family history of cardiovascular disease. The reference category is excellent self-rated health. HR indicates hazard ratio. ^a^*P* < .001 between sex and self-rated health status. ^b^*P* = .046 between sex and self-rated health status. ^c^*P* < .001 between sex and self-rated health status. ^d^*P* = .009 between sex and self-rated health status.

The rates of death and the components of the primary outcome are presented in Figures 1 and 3, respectively. Rates of each outcome increased with declining self-rated health. After adjustment, results remained consistent. Poorer self-rated health was associated with a higher rate of outcomes, with greater HRs for fair to poor health for women compared with men for myocardial infarction (1.77 [95% CI, 1.34-2.34] vs 0.99 [95% CI, 0.80-1.22]; *P* < .001), heart failure (3.94 [95% CI, 2.35-6.58] vs 2.30 [95% CI, 1.57-3.36]; *P* = .046), cardiovascular death (2.61 [95% CI, 2.11-3.23] vs 1.89 [95% CI, 1.58-2.25]; *P* < .001) and all-cause death (2.41 [95% CI, 2.11-2.74] vs 2.06 [95% CI, 1.83-2.32]; *P* = .009) (Figure 4). When using 5 self-rated health categories in women, the adjusted HR was 1.11 (95% CI, 0.97-1.26) for very good health, 1.50 (95% CI, 1.32-1.72) for good health, 2.01 (95% CI, 1.73-2.34) for fair health, and 3.12 (95% CI, 2.56-3.79) for poor health. For men, the corresponding HRs were 0.99 (95% CI, 0.90-1.10) for very good health, 1.14 (95% CI, 1.02-1.26) for good health, 1.42 (95% CI, 1.25-1.61) for fair health, and 2.20 (95% CI, 1.82-2.63) for poor heath, with greater HRs for women than men for each category (*P* < .001). Finally, when excluding individuals with anxiety or depression, the results remained consistent; in women compared with men, the HRs were higher for very good to good self-rated health (1.26 [95% CI, 1.10-1.44] vs 1.04 [95% CI, 0.94-1.14]) and fair to poor self-rated health (2.15 [95% CI, 1.82-2.56] vs 1.50 [95% CI, 1.31-1.71]) (*P* < .001).

## Discussion

In this large and contemporary primary prevention cohort, nearly 1 in 10 individuals rated their general state of health as fair to poor. We noted that the relative association of CVD outcomes with rating one’s own health as fair to poor resulted in greater hazards in women than men. In women, fair to poor self-rated health was associated with a 108% higher relative rate of CVD compared with excellent self-rated health, whereas in men there was a 45% relative increase in the rate of CVD. This difference persisted after adjustment for established markers of CVD such as traditional risk factors and enhancers, lifestyle and behavioral factors, social determinants of health, geographic residence, and a family history of CVD. Our results add to a growing body of literature that support self-rated health as an easily attainable, reliable, and reproducible patient-centric measure that is independently associated with the development of CVD.

Self-rated health has been linked to mortality in older epidemiologic studies conducted around the world.^10^ Its association, however, with the development of CVD continues to evolve. Epidemiologic studies as well as a post hoc analysis of a clinical trial population have suggested that poor self-rated health is associated with myocardial infarction,^43^ stroke,^44^ atrial fibrillation,^45^ and CVD,^46^ independent of traditional risk factors. Not only did we corroborate previous findings, we also undertook a comprehensive adjustment for other confounders to mitigate bias. Using a contemporary cohort where baseline self-rated health was reported in more than 98% of individuals, we found that the association was attenuated, but not fully explained, by cardiovascular risk factors as well as behavioral and lifestyle factors. Social determinants of health as well as ethnocultural factors had smaller additional impact on the association.^47^ Even adjustment for residence, which is strongly linked to health care access, use of services, and how individuals endorse their health, did not explain the association between self-rated health and CVD, supporting its robustness as a marker of cardiovascular risk.^48^

Whether the prognostic importance of self-rated health varies by sex remains the subject of debate. Some studies have suggested that the mortality risk with poor self-rated health is higher in men rather than women, while others have shown the opposite, with a paucity of data examining sex differences related to the development of CVD.^49,50,51^ We found that poorer self-rated health was associated with a greater rate of CVD in women than men, consistent with many physical, social, and psychological determinants of health.^14,16,17,18,19,52,53^ Explanations for this finding are not readily apparent. Women’s assessment of their health is often more accurate and concordant with symptoms and physical limitations, which may strengthen its association relative to men.^54^ It is plausible, on the other hand, that men may overestimate their health, thereby weakening its apparent association with outcomes. While some authors have suggested men may be more likely to accurately report the severity of risk factors, implying less residual risk explained by self-rated health after adjustment,^55^ our models were adjusted for conditions obtained from self-report, validated administrative databases, and objective laboratory measurements, which would mitigate this effect. Finally, it is conceivable that this simple self-assessment encapsulates total well-being, sense of belonging, and other holistic measures of health that are more relevant for CVD risk in women than men.^56,57^

Incorporating self-rated health into primary prevention assessments is advantageous for many reasons. It is reproducible, reliable, and easily implemented into clinical assessments with little added time or costs to clinicians, patients, or health systems. The underlying reason for individual ratings may not be entirely clear or modifiable in the traditional sense (eg, using antihypertensive agents to lower blood pressure) because it is a by-product of complex interactions between disease states and social factors (eg, social security, community support, health care access). This, nonetheless, does not invalidate its potential usefulness as a marker of CVD risk. In fact, one’s rating may be the culmination of the unmeasurable impact of markers that are not captured by any single laboratory (eg, low-density lipoprotein cholesterol level), physiologic (eg, systolic blood pressure), behavioral (eg, smoking), social (eg, financial stability), or psychological (eg, depression) measurement. Arguably, self-rated health better encapsulates the individual’s perspective of their well-being with less potential for inequitable treatment allocation, compared with nonmodifiable social constructs recommended by some guidelines in risk assessments (eg, race and ethnicity).^5,6,8,58^ Furthermore, numerous physical, psychological, and community-based interventions may improve well-being and self-rated health.^59,60^ Previous studies have demonstrated that self-rated health retains prognostic value independently of estimated atherosclerotic CVD risk using risk equations^61^; however, whether inclusion of self-rated health improves risk reclassification across treatment categories remains an avenue for future research.

### Limitations

Several limitations of our study are worth noting. First, enrollment in the OHS was contingent on individuals having sufficient proficiency in English or French to complete the baseline survey. This may limit generalizability to individuals who do not speak these languages. However, we performed extensive adjustment for racial and ethnic groups and immigration status to reduce confounding. Second, some confounders were based on self-report from the OHS. This may be subject to recall bias, resulting in a lower prevalence of risk factors between sexes. However, when feasible, validated administrative algorithms for case definitions of chronic diseases and laboratory data were used for adjustment. Third, we excluded individuals with prior CVD based on validated administrative algorithms capturing a CVD from prior hospitalization episodes. As such, patients with CVD diagnosed in outpatient settings and that did not result in a hospitalization were not excluded, which could have led to some misclassification. Fourth, our data were subject to missingness; however, the use of multiple imputation reduces the potential bias present in complete case analysis, with more accurate estimation of standard errors and preservation of statistical power.^39^ Fifth, results from the OHS may not be generalizable to all residents of Canada or other countries, where perception of health may be tied to differences in social factors such as living conditions or access to care.^62^ However, our sequential adjustment suggests the effect estimates for CVD are attenuated more so by cardiovascular risk factors and behavioral and lifestyle factors, and less so by social determinants. Sixth, despite extensive adjustment, it is possible the difference in the association between women and men may be due to the presence of unmeasured confounders.

## Conclusions

In this cohort study, 1 in 10 individuals without known CVD had fair to poor self-rated health. Poorer self-rated health demonstrated an independent association with the development of new CVD, with a greater relative hazard in women compared with men. These findings support the inclusion of self-rated health as a sex-specific and patient-centered measure of future risk to guide preventative treatment strategies.

## Notes

Supplement 1. **eTable 1.** Data Sources and Definitions **eTable 2.** Baseline Characteristics Across Self-Rated Health **eFigure 1.** Proportion With Missing Data **eFigure 2.** Cohort Creation **eFigure 3.** Time to Cardiovascular Disease Events

Supplement 2.
Data Sharing Statement
