# Supplementary material for: Sex Differences in Self-Rated Health and Cardiovascular Disease Events
Source: JAMA Netw Open. 2026 Apr 1;9(4):e264129. doi: 10.1001/jamanetworkopen.2026.4129 (PMC13044666; doi:10.1001/jamanetworkopen.2026.4129)
Supplement: Supplement 1. — eTable 1. Data Sources and Definitions eTable 2. Baseline Characteristics Across Self-Rated Health eFigure 1. Proportion With Missing Data eFigure 2. Cohort Creation eFigure 3. Time to Cardiovascular Disease Events [file jamanetwopen-e264129-s001.pdf]

Supplementary Online Content

Sud M, Qui F, Haldenby O, et al. Sex differences in self-rated health and cardiovascular disease events. *JAMA Netw Open.* 2026;9(4):e264129. doi:10.1001/jamanetworkopen.2026.4129

eTable 1. Data Sources and Definitions
eTable 2. Baseline Characteristics Across Self-Rated Health
eFigure 1. Proportion With Missing Data
eFigure 2. Cohort Creation
eFigure 3. Time to Cardiovascular Disease Events

This supplementary material has been provided by the authors to give readers additional information about their work.

© 2026 Sud M et al. *JAMA Network Open*.

eTable 1. Data Sources and Definitions

Characteristics	Source	Administrative Code
Myocardial Infarction	Linkage to Population-level Administrative Database	ICD9: 410<br>ICD10: I21, I22
Stroke	Linkage to Population-level Administrative Database	ICD9: 430, 431, 434, 436, 362.3<br>ICD10: I60, I61, I63 (excluding I63.6), I64, H34.1
Heart Failure	Linkage to Population-level Administrative Database	ICD9: 428<br>ICD10: I50
Coronary Revascularization	Linkage to Population-level Administrative Database	CCP: 4802, 4803, 4809<br>CCI: 1IJ50, 1IJ57GQ, 1IJ76
Peripheral Arterial Disease	Linkage to Population-level Administrative Database	ICD9: 441.3, 441.4, 440.2, 443.9, 444.2, CCP: 5012<br>ICD10: I70.2, I73.9, I71.3, I71.4, I70.2, I73.9, I74.3, I74.4, E115.0, E115.1 CCI: IJE57, IJE50, IJE87
Atrial Fibrillation	Linkage to Population-level Administrative Database	ICD9: 427.31,427.32<br>ICD10: I48
Receipt of Chemotherapy in the 1 year prior	Self-report (OHS Baseline Questionnaire)	NA
Metastatic Cancer	Linkage to Population-level Administrative Database	ICD9: 196, 197, 198, 199<br>ICD10:C77, C78, C79, C80
Liver Disease	Linkage to Population-level Administrative Database	ICD9:456.0, 456.1, 456.2<br>ICD10:I85.0, I85.9, I86.4, I98.2, K70.4, K71.1, K72.1, K72.9, K76.5, K76.6, K76.7
Dementia	Linkage to Population-level Administrative Database	i) at least 3 OHIP claims with a dementia diagnosis (OHIP: 290, 331, 797) which are at least 30 days apart within a 2 year period; or<br>ii) at least 1 hospitalization or day surgery with a dementia diagnosis record (ICD-10: F00.0, F00.1, F00.2, F00.9, F01.0, F01.1, F01.2, F01.3, F01.8, F01.9, F02.0, F02.1, F02.2,F02.3, F02.4, F02.8, F03.x,

© 2026 Sud M et al. *JAMA Network Open*.

Characteristics	Source	Administrative Code
		F05.1, F06.5, F06.6, F06.8, F06.9, F09.x, G30.0, G30.1, G30.8, G30.9, G31.0 G31.1,R54.x); or<br>iii) one Ontario Drug Benefit Database claim for a cholinesterase inhibitor in the prior 5 years.
Dialysis dependency	Linkage to Population-level Administrative Database	At least 2 separated by at least 90 days, but less than 150 days.<br><br>OHIP service codes: R849, G323, G325, G326, G332*, G860, G862, G865, G863, G866, G330, G331, G333, G861, G082, G083, G085, G090, G091, G092, G093, G094, G095, G096, G294, G295, G864, H540, H740<br>CCI procedure codes: 5195, 6698<br>CCP procedure code: 1PZ21<br>*For G332, exclude records on or after 2008
Nursing Home Resident	Linkage to Population-level Administrative Database	Long-term care flag in the Ontario Drug Benefit Database
High Blood Cholesterol	Self-report (OHS Baseline Questionnaire)	NA
Diabetes	Linkage to Population-level Administrative Database	i) one hospital admission with a diabetes diagnosis (ICD-9: 250x; ICD-10: E10, E11, E13, E14) in the past 1 year; or<br>ii) two OHIP claim with a diabetes diagnosis (OHIP: 250); or<br>iii) one claim in the Ontario Drug Benefit Database for a diabetes-specific medication in the past year
Hypertension	Linkage to Population-level Administrative Database	i) one hospital admission or same-day surgery record with a hypertension diagnosis (ICD-9: 401x, 402x, 403x, 404x, 405x; ICD-10: I10, I11, I12, I13, I15); or<br>ii) an OHIP claim with a hypertension diagnosis followed by either an OHIP claim or a hospital admission / same day surgery record with a hypertension diagnosis within two years (OHIP: 401, 402, 403, 404, or 405).

© 2026 Sud M et al. *JAMA Network Open*.

Characteristics	Source	Administrative Code
		*Gestational hypertension is defined by a hypertension record 120 days before and up to 180 days after a hospital admission for delivery and is excluded.
Obstructive Sleep Apnea	Physical Measurements (OHS Baseline Assessment)	NA
Elevated Waist to Hip Ratio	Self-report (OHS Baseline Questionnaire)	NA
Depression	Self-report (OHS Baseline Questionnaire)	NA
Anxiety	Self-report (OHS Baseline Questionnaire)	NA
Rheumatoid Arthritis	Self-report (OHS Baseline Questionnaire)	NA
Lupus	Self-report (OHS Baseline Questionnaire)	NA
Psoriasis	Self-report (OHS Baseline Questionnaire)	NA
Total Cholesterol	Linkage to Population-level Laboratory Database	LOINC: 14647-2
High-density Lipoprotein Cholesterol	Linkage to Population-level Laboratory Database	LOINC: 14646-4
Triglycerides	Linkage to Population-level Laboratory Database	LOINC: 14927-8
Hemoglobin A1c	Linkage to Population-level Laboratory Database	LOINC: 17855-8, 17856-6, 41995-2, 4548-4, 71875-9
Fasting Glucose	Linkage to Population-level Laboratory Database	LOINC: 14771-0
Serum Creatinine	Linkage to Population-level Laboratory Database	LOINC: 14682-9
Systolic Blood Pressure	Physical Measurements (OHS Baseline Assessment)	NA
Body Mass Index	Physical Measurements (OHS Baseline Assessment)	NA
Smoking History	Self-report (OHS Baseline Questionnaire)	NA
Servings of Fruits and Vegetables / day	Self-report (OHS Baseline Questionnaire)	NA
Hours of Sleep / day	Self-report (OHS Baseline Questionnaire)	NA
Physical Activity Level	Self-report (OHS Baseline Questionnaire)	NA

© 2026 Sud M et al. *JAMA Network Open*.

Characteristics	Source	Administrative Code
Race/ethnicity	Self-report (OHS Baseline Questionnaire)	**Black**: "Black (African or Caribbean descent)" response<br>**East Asian**: "Chinese" or "Korean" or "Japanese" response<br>**Latin**: "Latin American/Hispanic" response<br>**Middle Eastern**: "Arab (e.g. Egypt, Iraq, Jordan, Lebanon)" or "West Asian (e.g. Turkey, Iran, Afghanistan)" response<br>**South Asian**: "South Asian (e.g. India, Sri Lanka, Pakistan, Bangladesh)" response<br>**Southeast Asian**: "Southeast Asian (e.g. Malaysia, Indonesia, Vietnam, Cambodia, Loas)" or "Filipino" response<br>**White**: "White (European descent)" response<br>**Multiracial**: when any 2 or more responses selected<br>**Other**: "Other", "Jewish" or "Aboriginal (First Nations, Métis, Inuit)" response
Recent Immigrant	Linkage to Population-level Administrative Database	Landing date from 1985 onwards in the Immigration, Refugees, and Citizenship Canada (IRCC)'s Permanent Resident Database
Yearly Income	Self-report (OHS Baseline Questionnaire)	NA
Education Level	Self-report (OHS Baseline Questionnaire)	NA
Employment	Self-report (OHS Baseline Questionnaire)	NA
Marital Status	Self-report (OHS Baseline Questionnaire)	NA

© 2026 Sud M et al. *JAMA Network Open*.

Characteristics	Source	Administrative Code
Cardiovascular Health Regions	Linkage to Population-level Administrative Database	Local Health Integration Networks determined through the Postal CodeOM Conversion File<br><br>High Risk Local Health Integration Network: North East, North Simcoe Muskoka, North West, Erie St. Claire<br><br>Intermediate Risk Local Health Integration Network: Champlain, South East, Central East, Central West, Waterloo Wellington, Hamilton Niagra Hadimand Brant, South West<br><br>Low Risk Local Health Integration Network: Central, Toronto Central, Mississauga Halton
Rural Residence	Linkage to Population-level Administrative Database	Community size <= 10,000 in the Postal CodeOM Conversion File
Family History of Cardiovascular Disease	Self-report (OHS Baseline Questionnaire)	NA

© 2026 Sud M et al. *JAMA Network Open*.

eTable 2. Baseline Characteristics Across Self-Rated Health

Characteristics	Proportion %, (95% Confidence Interval)				
		Individual's Self-Rated Health			
	Total	Excellent	Very Good to Good	Fair to Poor	p-value
	N=170,197	N=28,471	N=123,684	N=18,042	
**Demographics**					
Age, yrs, median (IQR)	48 (36-58)	48 (36-58)	48 (35-58)	49 (38-58)	<.0001
Female	61.6% (61.3% - 61.8%)	60.8% (60.2% - 61.4%)	61.3% (61.0% - 61.6%)	64.6% (63.9% - 65.3%)	<.0001
**Traditional Risk Factors**					
High Blood Cholesterol	19.0% (18.8%-19.2%)	11.0% (10.6%-11.4%)	19.1% (18.9%-19.4%)	30.7% (30.0%-31.4%)	<.0001
Diabetes	7.6% (7.5% - 7.7%)	2.1% (1.9% - 2.3%)	7.0% (6.9% - 7.1%)	20.4% (19.9% - 21.0%)	<.0001
Hypertension	21.9% (21.7% - 22.1%)	11.5% (11.2% - 11.9%)	22.2% (22.0% - 22.4%)	36.0% (35.3% - 36.7%)	<.0001
Depression	6.6% (6.4%-6.7%)	3.0% (2.7%-3.2%)	6.2% (6.0%-6.3%)	14.8% (14.2%-15.4%)	<.0001
Anxiety	11.4% (11.3%-11.6%)	4.4% (4.1%-4.6%)	10.4% (10.2%-10.6%)	29.5% (28.9%-30.2%)	<.0001
Rheumatoid Arthritis	10.7% (10.3%-11.1%)	4.6% (4.1%-5.0%)	10.0% (9.5%-10.4%)	25.3% (24.5%-26.1%)	<.0001
Lupus	3.3% (3.2%-3.4%)	1.3% (1.1%-1.4%)	3.0% (2.9%-3.1%)	8.4% (8.0%-8.9%)	<.0001
Psoriasis	0.4% (0.3%-0.4%)	0.1% (0.1%-0.1%)	0.3% (0.2%-0.3%)	1.5% (1.4%-1.7%)	<.0001
Obstructive Sleep Apnea	6.6% (6.4%-6.7%)	3.0% (2.7%-3.2%)	6.2% (6.0%-6.3%)	14.8% (14.2%-15.4%)	<.0001
Elevated Waist to Hip Ratio	61.8% (61.5%-62.1%)	54.0% (53.3%-54.7%)	62.4% (62.1%-62.8%)	69.8% (68.9%-70.6%)	<.0001
Total Cholesterol, mg/dL, median (IQR)	190 (164-216)	192 (167-218)	189 (163-216)	187 (159-215)	<.0001
High-density Lipoprotein Cholesterol, mg/dL, median (IQR)	56 (46-67)	61 (50-72)	56 (45-67)	51 (41-62)	<.0001
Triglycerides, mg/dL, median (IQR)	110 (68-162)	91 (55-140)	111 (69-162)	137 (91-192)	<.0001
Hemoglobin A1c, %, median (IQR)	5.6 (5.2-6.1)	5.5 (5.1-6.0)	5.6 (5.2-6.1)	5.9 (5.4-6.5)	<.0001
Fasting Glucose, mmol/L, median (IQR)	5.1 (4.5-5.9)	5.0 (4.3-5.6)	5.1 (4.5-5.9)	5.5 (4.7-6.5)	<.0001
Estimated Glomerular Filtrate Rate, median (IQR), mL/min	97 (85-109)	96 (84-108)	97 (85-109)	97 (85-108)	<.0001
Systolic Blood Pressure, mmHg, median (IQR)	117 (106-128)	115 (104-125)	117 (106-128)	119 (108-130)	<.0001

© 2026 Sud M et al. *JAMA Network Open*.

Characteristics	Proportion %, (95% Confidence Interval)				
		**Individual's Self-Rated Health**			
	Total	Excellent	Very Good to Good	Fair to Poor	p-value
	N=170,197	N=28,471	N=123,684	N=18,042	
BMI ≥ 30 kg/m^2, n(%)	23.5% (23.3%-23.7%)	9.5% (9.1%-9.9%)	23.8% (23.5%-24.0%)	43.9% (43.1%-44.7%)	<.0001
**Lifestyle and Behavior Factors**					
Active Daily Smoker	10.6% (10.4%-10.7%)	4.3% (4.1%-4.5%)	10.4% (10.3%-10.6%)	21.3% (20.7%-21.9%)	<.0001
≥5 Servings of Fruits and Vegetables / day	46.3% (46.1%-46.6%)	58.4% (57.8%-59.0%)	45.3% (45.0%-45.6%)	34.4% (33.7%-35.1%)	<.0001
7 to < 9 hours of Sleep / day	64.4% (64.2%-64.6%)	72.2% (71.7%-72.8%)	64.9% (64.7%-65.2%)	48.4% (47.6%-49.2%)	<.0001
Low Physical Activity Level	29.0% (28.8%-29.3%)	16.8% (16.3%-17.3%)	29.1% (28.8%-29.3%)	48.2% (47.4%-49.0%)	<.0001
**Social Determinants**					
Race/ethnicity					
Black	1.3% (1.3%-1.4%)	1.5% (1.4%-1.6%)	1.3% (1.2%-1.3%)	1.5% (1.3%-1.7%)	<.0001
East Asian	4.1% (4.0%-4.2%)	3.6% (3.3%-3.8%)	4.2% (4.1%-4.3%)	4.1% (3.8%-4.4%)	
Latin	0.8% (0.7%-0.8%)	0.7% (0.6%-0.9%)	0.8% (0.7%-0.8%)	0.8% (0.7%-0.9%)	
Middle Eastern	0.9% (0.8%-0.9%)	0.9% (0.8%-1.0%)	0.9% (0.8%-0.9%)	0.9% (0.7%-1.0%)	
South Asian	3.4% (3.3%-3.5%)	3.3% (3.1%-3.6%)	3.4% (3.3%-3.5%)	3.5% (3.3%-3.8%)	
Southeast Asian	1.1% (1.0%-1.1%)	1.0% (0.9%-1.1%)	1.1% (1.0%-1.1%)	1.2% (1.1%-1.4%)	
White	80.9% (80.7%-81.1%)	83.0% (82.5%-83.4%)	81.0% (80.7%-81.2%)	77.1% (76.5%-77.8%)	
Multiracial	4.5% (4.4%-4.6%)	3.7% (3.4%-3.9%)	4.5% (4.4%-4.6%)	6.1% (5.7%-6.4%)	
Other	3.0% (2.9%-3.1%)	2.3% (2.2%-2.5%)	2.9% (2.8%-3.0%)	4.7% (4.4%-5.1%)	
Recent Immigrant	8.2% (8.1% - 8.3%)	8.1% (7.8% - 8.4%)	8.2% (8.1% - 8.4%)	8.3% (7.9% - 8.7%)	0.8347
Yearly Income < $50,000CDN / year	27.7% (27.4%-27.9%)	19.8% (19.3%-20.3%)	27.1% (26.8%-27.4%)	43.8% (43.1%-44.6%)	<.0001
University Bachelors or Graduate Education Level	44.5% (44.3%-44.7%)	57.3% (56.8%-57.9%)	44.0% (43.8%-44.3%)	27.6% (26.9%-28.2%)	<.0001
Actively Employed	32.4% (32.2%-32.7%)	28.1% (27.6%-28.6%)	31.1% (30.9%-31.4%)	48.3% (47.5%-49.0%)	<.0001
**Marital Status**					

© 2026 Sud M et al. *JAMA Network Open*.

Characteristics	Proportion %, (95% Confidence Interval)				
		**Individual's Self-Rated Health**			
	Total N=170,197	Excellent N=28,471	Very Good to Good N=123,684	Fair to Poor N=18,042	p-value
Divorced	7.6% (7.5%-7.7%)	7.2% (6.9%-7.5%)	7.3% (7.1%-7.4%)	10.7% (10.3%-11.2%)	<.0001
Married and or living with partner	67.4% (67.2%-67.6%)	69.6% (69.1%-70.1%)	68.1% (67.8%-68.3%)	59.3% (58.6%-60.0%)	
Separated	3.9% (3.8%-4.0%)	3.5% (3.3%-3.7%)	3.7% (3.6%-3.8%)	5.8% (5.5%-6.2%)	
Single or never married	18.3% (18.1%-18.5%)	17.2% (16.8%-17.6%)	18.2% (18.0%-18.4%)	20.8% (20.2%-21.4%)	
Widowed	2.8% (2.7%-2.9%)	2.5% (2.3%-2.7%)	2.8% (2.7%-2.9%)	3.3% (3.0%-3.6%)	
**Residence**					
Residing in a High Cardiovascular Disease Risk Region	14.0% (13.8%-14.1%)	12.1% (11.8%-12.5%)	14.0% (13.8%-14.2%)	16.5% (16.0%-17.0%)	<.0001
Rural Residence	9.6% (9.4%-9.7%)	8.6% (8.3%-8.9%)	9.7% (9.5%-9.9%)	10.0% (9.6%-10.4%)	<.0001
**Genetic Factors**					
Family History of Cardiovascular Disease	39.9% (39.7%-40.1%)	35.3% (34.8%-35.9%)	39.7% (39.4%-40.0%)	48.6% (47.9%-49.4%)	<.0001

Abbreviations: BMI, body mass index (calculated as weight in kilograms 1 divided by height in meters squared); CAD$, Canadian dollars; CVD, cardiovascular disease; HbA1c, hemoglobin A1c. SI conversion factors: To convert cholesterol to mmol/L, multiply by 0.0259; glucose to 5 mmol/L, multiply by 0.0555; HbA1c to a proportion of total hemoglobin, multiply by 0.01; triglycerides to mmol/L, multiply by 0.0113.

[a]Results are reported after multiple imputation and pooled across 30 datasets

© 2026 Sud M et al. *JAMA Network Open*.

eFigure 1. Proportion With Missing Data

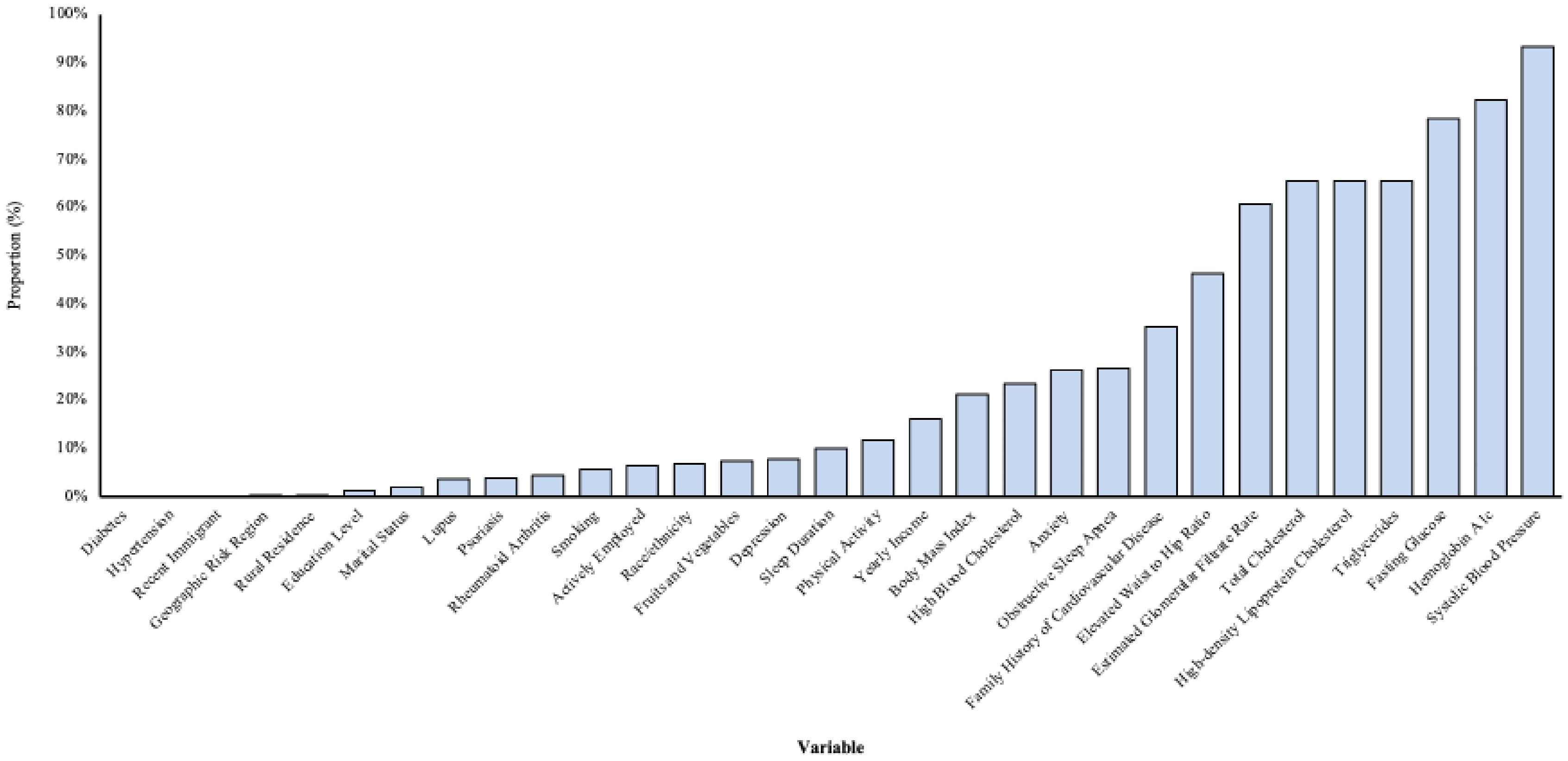

© 2026 Sud M et al. *JAMA Network Open*.

eFigure 2. Cohort Creation

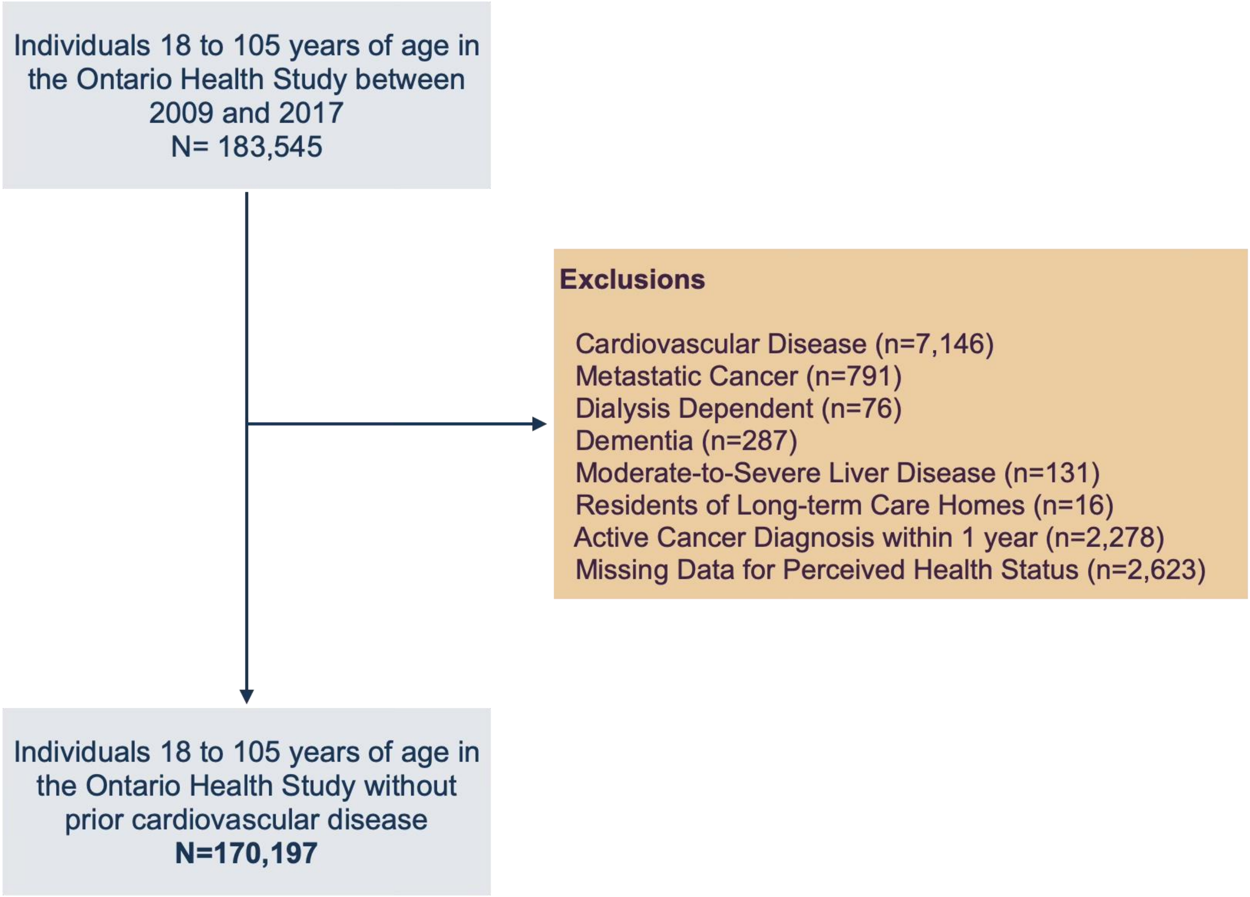

© 2026 Sud M et al. *JAMA Network Open*.

eFigure 3. Time to Cardiovascular Disease Events

Cumulative incidence curves depict time to cardiovascular disease events in women and men across excellent (green line), very good to good (red line) and fair to poor (blue line) self-rated health. Gray's test was significant with p< 0.001 in women and men.

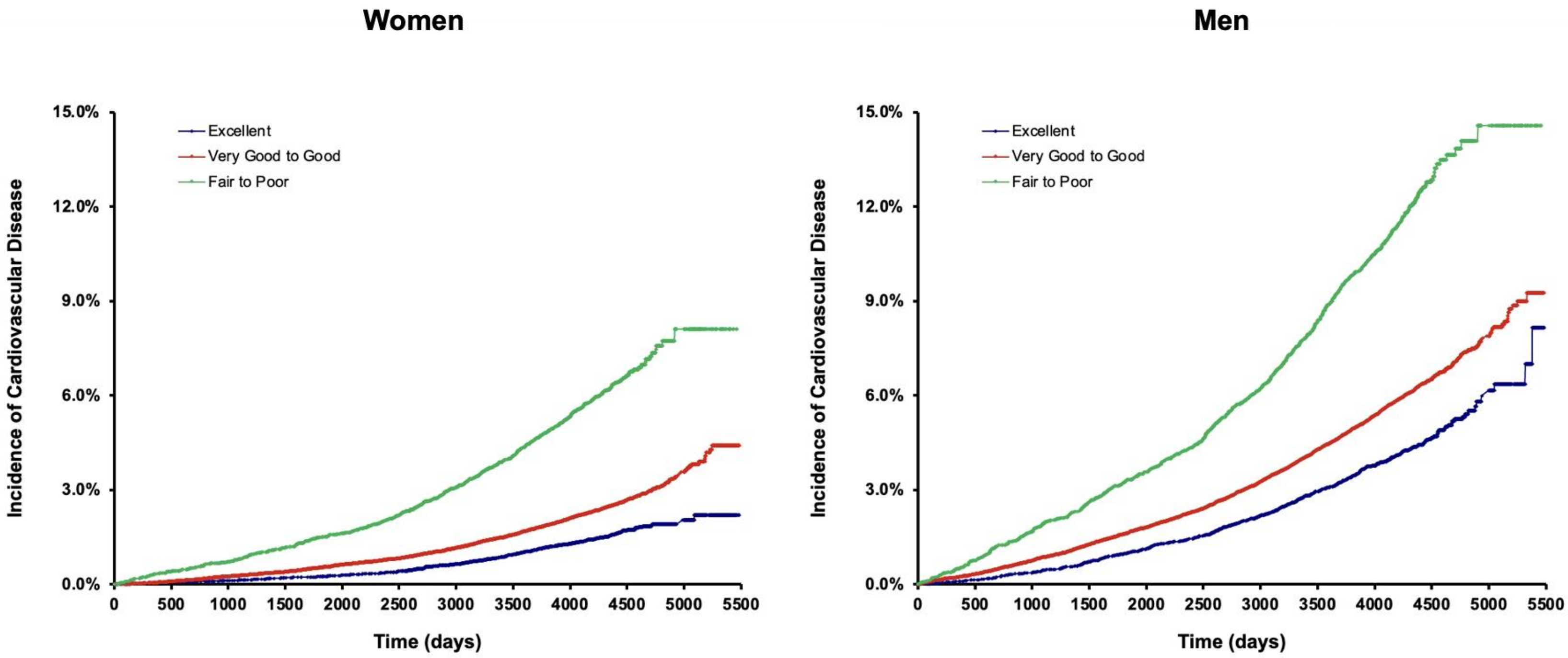

© 2026 Sud M et al. *JAMA Network Open*.
