# Supplementary material for: Sex Differences in Self-Rated Health and Cardiovascular Disease Events
Source: JAMA Netw Open. 2026 Apr 1;9(4):e264129. doi: 10.1001/jamanetworkopen.2026.4129 (PMC13044666; doi:10.1001/jamanetworkopen.2026.4129)
Supplement: Supplement 2. — Data Sharing Statement [file jamanetwopen-e264129-s002.pdf]

Data Sharing Statement

Sud. Sex Differences in Self-Rated Health and Cardiovascular Disease Events. *JAMA Netw Open*. Published April 01, 2026. doi:10.1001/jamanetworkopen.2026.4129

Data
Data available: No
